# A remote monitoring and telephone nurse coaching intervention to reduce readmissions among patients with heart failure: study protocol for the Better Effectiveness After Transition - Heart Failure (BEAT-HF) randomized controlled trial

**DOI:** 10.1186/1745-6215-15-124

**Published:** 2014-04-13

**Authors:** Jeanne T Black, Patrick S Romano, Banafsheh Sadeghi, Andrew D Auerbach, Theodore G Ganiats, Sheldon Greenfield, Sherrie H Kaplan, Michael K Ong

**Affiliations:** 1 Resource and Outcomes Management Department, Cedars-Sinai Health System, Los Angeles, CA 90048, USA; 2 Department of Medicine, University of California, Davis, Sacramento, USA; 3 Department of Medicine, University of California, San Francisco, USA; 4 Department of Family and Preventive Medicine, University of California, San Diego, USA; 5 Department of Medicine, University of California, Irvine, USA; 6 Department of Medicine, University of California, Los Angeles, USA

**Keywords:** Heart failure, Telemonitoring, Nurse coaching, Readmission, Care coordination, Self-care

## Abstract

**Background:**

Heart failure is a prevalent health problem associated with costly hospital readmissions. Transitional care programs have been shown to reduce readmissions but are costly to implement. Evidence regarding the effectiveness of telemonitoring in managing the care of this chronic condition is mixed. The objective of this randomized controlled comparative effectiveness study is to evaluate the effectiveness of a care transition intervention that includes pre-discharge education about heart failure and post-discharge telephone nurse coaching combined with home telemonitoring of weight, blood pressure, heart rate, and symptoms in reducing all-cause 180-day hospital readmissions for older adults hospitalized with heart failure.

**Methods/Design:**

A multi-center, randomized controlled trial is being conducted at six academic health systems in California. A total of 1,500 patients aged 50 years and older will be enrolled during a hospitalization for treatment of heart failure. Patients in the intervention group will receive intensive patient education using the ‘teach-back’ method and receive instruction in using the telemonitoring equipment. Following hospital discharge, they will receive a series of nine scheduled health coaching telephone calls over 6 months from nurses located in a centralized call center. The nurses also will call patients and patients’ physicians in response to alerts generated by the telemonitoring system, based on predetermined parameters. The primary outcome is readmission for any cause within 180 days. Secondary outcomes include 30-day readmission, mortality, hospital days, emergency department (ED) visits, hospital cost, and health-related quality of life.

**Discussion:**

BEAT-HF is one of the largest randomized controlled trials of telemonitoring in patients with heart failure, and the first explicitly to adapt the care transition approach and combine it with remote telemonitoring. The study population also includes patients with a wide range of demographic and socioeconomic characteristics. Once completed, the study will be a rich resource of information on how best to use remote technology in the care management of patients with chronic heart failure.

**Trial registration:**

ClinicalTrials.gov # NCT01360203.

## Background

Heart failure is a prevalent and costly condition, affecting some 5.1 million people in the U.S. [1]. It accounts for more than 1 million hospitalizations and approximately 2.8 million physician office, emergency department (ED), and hospital outpatient visits each year, at an estimated cost exceeding $32 billion [2]. Heart failure is the most common reason for both hospitalization and readmission among Medicare beneficiaries [3]. The Centers for Medicare & Medicaid Services (CMS) has publicly reported hospital readmission rates for Medicare beneficiaries with heart failure on its Hospital Compare website since 2009. The national 30-day heart failure readmission rate for the period July 2009 to June 2012 was 23.1% [4], with substantial variation among hospitals [5,6]. Excess 30-day readmissions for Medicare patients discharged with three targeted conditions, including heart failure, now trigger a financial penalty through the Hospital Readmission Reduction Program (HRRP) mandated by the Patient Protection and Affordable Care Act [7].

Estimates of the proportion of readmissions that are potentially avoidable vary widely [8]. However, the fact that 52% of Medicare patients readmitted within 30 days of a heart failure discharge had not seen an outpatient provider [3] has focused attention on patients’ transition from the hospital - where they are primarily passive recipients of care - to home, where they must be responsible for their own care. Interventions to improve the care transition process have been shown to prevent readmissions while potentially improving morbidity and mortality in randomized controlled trials, including the Transitional Care Program [9,10] and the Care Transition Intervention [11]. However, these approaches require face-to-face interaction with patients in their homes. This makes them costly and difficult to sustain in the current hospital and physician reimbursement environment, which remains predominantly fee-for-service [12]. A less costly but still effective care transition intervention would assist hospitals as they strive to redesign their services in anticipation of different payment models, such as bundled payments and Accountable Care Organizations, mandated by the Affordable Care Act.

Many stakeholders are hoping that technology can be a cost-effective substitute for in-person transition coaches, allowing early intervention to prevent subsequent healthcare utilization. Some remote telehealth programs use standard telephones to deliver education and communicate patient reports of self-monitoring, while others use telemonitoring devices that transmit physiologic data using digital, wireless, or Bluetooth technology. However, evidence has been mixed regarding the effectiveness of these programs for patients with heart failure. Inglis et al.’s 2011 Cochrane Review conducted a meta-analysis of studies using standard telephones (referred to as ‘structured telephone support’) or telemonitoring devices for patients with heart failure, based on study results published from 2006 through 2009. Both approaches were found to be effective in reducing the risk of heart failure-specific hospitalizations and all-cause mortality and also showed a small benefit for all-cause hospitalization [13,14]. Most of the included studies were relatively small. The meta-analysis did not include two larger, more recent multi-center studies, Telemonitoring to Improve Heart Failure Outcomes (TELE-HF, 1,653 patients) [15] or Telemedical Interventional Monitoring in Heart Failure (TIM-HF, 710 patients) [16]. The Tele-HF intervention had no effect on all-cause readmission or death within 180 days, and no significant effect on secondary endpoints such as hospitalization for HF, hospital days, or time to the primary endpoint. Tele-HF used an interactive voice response system that required the patient to enter daily weight and symptoms using the touchtone keys on a standard telephone. This lack of human contact may have been the reason for Tele-HF’s low adherence rate of 55% at the end of 6 months. The same investigators had conducted a small prior study that successfully reduced readmission rates among patients with heart failure using a nurse-based telehealth intervention, suggesting that the nurse role [17] and the optimal balance of human and technological resources [18] are critical. TIM-HF used wireless technology with a personal digital assistant, and had 81% adherence over a median of 26 months of follow-up. However, this study also found no effect on its primary endpoint, total mortality, or on the composite measure of cardiovascular mortality and heart failure hospitalization after 1 year [16]. Thus, the effectiveness of these programs remains unclear, and additional evidence is needed to understand why some interventions succeeded while others did not.

This study, Better Effectiveness After Transition - Heart Failure (BEAT-HF) has several important features. The intervention combines a telephonic adaptation of care transition programs with telemonitoring. It will be one of the largest randomized controlled trials of telemonitoring in heart failure, with a projected sample size of 1,500 patients. Patients will be enrolled at six sites throughout the state of California and will include a substantial proportion of racial/ethnic minority subjects and individuals with limited English proficiency. In addition to the standard outcomes of readmission, mortality, and hospital days, we will collect data to assess health literacy, depression, social support, informal caregiving, and medication adherence in addition to health-related quality of life (HRQOL).

The main objective of the BEAT-HF study is to evaluate the effectiveness of this remote care transition intervention in reducing all-cause 180-day hospital readmissions for older adults hospitalized with heart failure. We will also assess 30-day readmissions, all-cause mortality, hospital days, hospital costs, and HRQOL. Secondary objectives are to understand the influence of moderating variables such as socioeconomic status, health literacy, and patient co-morbidities, as well as the impact of the intervention on intermediate factors such as self-care knowledge and behavior and medication adherence.

## Methods/Design

### Design

BEAT-HF is a prospective, two-arm multi-center, randomized controlled trial being conducted at six academic health systems in California to compare usual care with a telehealth-based care transition intervention for older patients who are discharged home after inpatient treatment for decompensated heart failure. We plan to enroll 1,500 patients before hospital discharge, with half randomized to usual care and half to an education/nurse coaching/telemonitoring intervention. Randomization is stratified by medical center. The original BEAT-HF design was a three arm randomized controlled study comparing an adaptation of the Tele-HF protocol, telephone-based health coaching based on the Transitional Care model, and concurrent controls. However, the findings from the Tele-HF study led to a redesign, in which we decided to combine the care transition telephone coaching with telemonitoring.

The study has been approved by the UCLA Institutional Review Board (IRB). Approval at the other participating sites was obtained through a process in which an institutional IRB agrees to rely upon the review and approval of another IRB under the auspices of an established Memorandum of Understanding. The reliance procedure enabled the participating medical centers to avoid site-specific negotiations regarding the consent documents. The study is registered at ClinicalTrials.gov (NCT01360203).

### Setting and sample

The study is being conducted at six academic medical centers located throughout the state of California. Five of the medical centers are part of the University of California system; the sixth is Cedars-Sinai Medical Center in Los Angeles, which has a mixed model medical staff that includes full time faculty, a multi-specialty group practice, and a large number of independent private physicians. Three of the health systems are major heart transplant centers, and three serve as safety net hospitals for their respective regions. A large proportion of individuals with heart failure are covered by Medicare, the federal health insurance program for those who are aged 65 years or older or disabled, and/or the federal-state Medicaid program for the poor. Others may have employer-sponsored or individual commercial health insurance. All patients suffering acute exacerbation of heart failure receive inpatient hospital care in the U.S., regardless of insurance coverage. However, those who are uninsured may have difficulty accessing outpatient care.

As an effectiveness study, BEAT-HF seeks to enroll a broad range of patients hospitalized with heart failure. Individuals admitted as hospital inpatients or on observation status are eligible if they are aged 50 years or older, receiving active treatment for decompensated heart failure (defined as initiation of or an increase in diuretic treatment), and are expected to be discharged to their home. According to the U.S. Census Bureau, 43.8% of California residents aged 5 years and older in 2011 spoke a language other than English at home [19]. We began study enrollment with patients who spoke the two most common languages, English or Spanish. Our analysis of patients not enrolled initially due to language showed that the next most frequent languages spoken by patients with heart failure at the six medical centers were Farsi (Persian), Russian, and Cantonese/Mandarin. We expanded the study to enroll in both Persian and Russian, but did not extend to Cantonese/Mandarin or other languages due to costs of translation and interpreter services. All study materials, including consent forms, teaching and equipment reference materials, and surveys were translated to ensure that non-English speaking patients received the same information. Translation was performed by professional translators and incorporated appropriate cultural adaptation, as recommended by the IRB overseeing the study.

The study exclusions can be grouped into three main categories: (1) patients who do not have the cognitive or physical ability, or access to resources, required to participate fully in the BEAT-HF intervention; (2) patients already in a system of care that provides more health provider contacts than the planned intervention; or (3) patients whose heart failure is due to a cardiovascular condition that is expected to improve due to medical intervention. (See the detailed ‘List of exclusion criteria’).

### List of exclusion criteria

1) *Lacking ability or resources to participate in intervention*

■ Dementia (3 or more incorrect items of 6 on Callahan screener)

■ Inability to conduct telephone conversation despite use of assistive technology

■ No working telephone

■ Inability to stand on scale

■ Weight more than 450 pounds

■ No usual source of care and no provider to be assigned upon discharge (free clinic is acceptable)

■ Expected to be discharged to homeless shelter or other transitional housing lacking a secure place to store telemonitoring equipment

2) *In a system of care that provides more health provider contacts than study intervention*

■ Long-term residence in skilled nursing facility

■ Expected to be discharged to long-term stay at skilled nursing facility

■ Expected to be transferred to another acute care or rehabilitation hospital

■ On chronic hemodialysis

■ Solid organ transplant recipient or listed for transplant

■ Recipient of left ventricular assist device or planned to receive one

3) *Heart failure is due to a cardiovascular condition expected to improve with medical intervention*

■ Receiving percutaneous coronary intervention during current stay

■ Interventional valve procedure (surgery or transcatheter) planned during current admission

4) *Other exclusions*

■ Admitted with acute myocardial infarction (except demand ischemia)

■ Critical aortic stenosis (defined as an estimated valve area of <0.75 cm^2^) with no intervention planned

■ Surgery planned during current admission (cardiovascular or other)

■ On hospice or hospice-bound

■ Residence outside California

■ Currently participating in another telemonitoring program (for example, through a health plan or a Veterans Affairs clinic)

Multiple sources are used to screen for patients who potentially meet the study’s inclusion criteria. These include lists of patients with heart failure prepared by the hospitals’ Core Measures nurses, lists of patients admitted to cardiology services, pharmacy data on patients receiving intravenous diuretics, and review of admitting complaints, for example, shortness of breath, pedal edema. Most exclusions are identified through review of electronic medical records or confirmed with the attending physician or bedside nurse, prior to approaching patients in person. Study nurses at each site visit potentially eligible patients in their hospital rooms to explain the study and determine interest in participating. Consent is obtained prior to discharge; patients are given time to decide on participation and many choose to discuss the study with family and caregivers. All eligible patients are recruited, whenever possible, however, some are discharged from the hospital before they can be approached or before completing the consent process.

The six-item Callahan screener is used to evaluate cognitive ability to participate in the intervention [20]. If the patient answers three or more questions correctly, informed consent is obtained and the patient is enrolled. The study nurse administers the baseline survey and then randomizes the patient.

### Intervention

**Figure 1:**
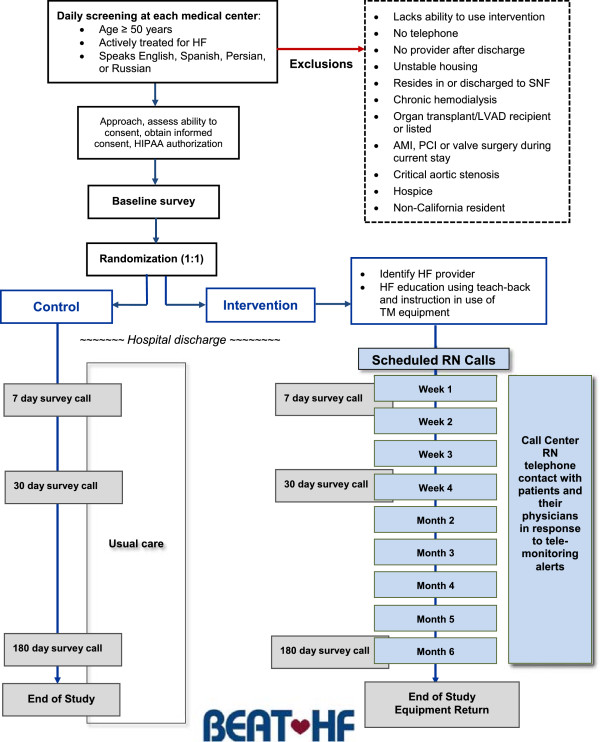
Diagram of BEAT-HF patient enrollment, randomization, intervention, and time line.

The BEAT-HF intervention consists of three components: pre-discharge heart failure education, regularly scheduled telephone coaching, and home telemonitoring of weight, blood pressure, heart rate, and symptoms, as illustrated in Figure 1. The pre-discharge health education is conducted by the study nurse, who is not part of the usual care team. The nurse guides patients through a booklet, *Caring for Your Heart: Living Well with Heart Failure*, that was developed for patients with low health literacy [21]. The topics covered in the booklet include an explanation of heart failure, medication adherence, salt avoidance, fluid monitoring, exercising with heart failure, daily check-up of weight and edema, and when to call the heart failure treatment team. The study nurse uses the ‘teach-back’ approach to ensure patient understanding. Family members or other caregivers are included in the teaching sessions if they are available and want to participate. The pre-discharge education also includes a demonstration of how to use the remote home telemonitoring equipment and an explanation of why monitoring physiologic parameters is important for patients.

The equipment consists of the FDA-approved Ideal Life Pod™, a Bluetooth-enabled wireless gateway, the Ideal Life Body-Manager (weight scale), and the Ideal Life BP-Manager, a blood pressure/heart rate monitor integrated with a device that displays text questions and sends simple text responses. From the user perspective, these measurement devices are similar to ordinary weight scales and blood pressure cuffs. The transmission pod is a simple plug and play device that can be placed anywhere within a home; it does not need to be in the immediate vicinity of the measurement devices. We reviewed several sets of devices from different manufacturers and made the selection based on ease of use and the company’s willingness to be responsible for all system components. These devices are similar to those used in previous telehealth studies [16,22].

The hospital-based nurse provides a ‘warm handoff’ to the centralized telephone nurse coaches by giving patients the name of the nurse who will be contacting them after discharge and showing her photo. Study nurses use interpreters when needed to communicate with patients who speak Spanish, Persian, or Russian. When interpreters are not available in person, a three-way telephone interpretation service is used. Patients take the assembled equipment home, along with a binder that includes the heart failure education booklet, a one-page summary of the study, a toll-free number for technical support by Ideal Life, a checklist for completion of the 7-day, 30-day, and 180-day telephone surveys, a copy of the upcoming 7-day survey, and copies of their consent and HIPAA authorization forms.

A call center nurse first contacts each enrolled patient 2 or 3 days after discharge from the hospital to reinforce the pre-discharge health coaching topics. Telephone nurse coaching then occurs on a weekly basis during the first month post-discharge. The call center nurses have access to patients’ medical histories and medication records contained in the electronic health records of each participating medical center. After the first month, nurse coaching calls are made monthly until the end of the 6-month study period. All intervention patients receive a minimum of nine scheduled telephone coaching calls, generally from the same nurse over time.

Intervention patients are asked to use the Ideal Life Body-Manager and BP-Manager daily to transmit their weight, blood pressure, heart rate, and responses to three symptom questions. These wireless devices can be placed anywhere in a patient’s home. They transmit information to the Ideal Life Pod, which retransmits the collected data via the cellular bandwidth to a secure server that is accessed daily by the centralized call center nurses. There are two groups of symptom questions that alternate daily, to reduce respondent burden and minimize repetition. Readings that exceed predetermined threshold parameters generate a trigger for the call center nurse, who telephones the patient to investigate. When symptoms are concerning, patients are encouraged to contact their providers. Table 1 shows the biometric parameters and symptom responses that prompt the nurse to contact the patient’s physician with an urgent alert. The biometric parameters can be changed by the physician. If deemed necessary, the call center nurses will advise patients to call 911 or go to their nearest hospital emergency room. A call center nurse also calls any patient who has stopped transmitting data to determine the reason and encourage the patient to resume daily monitoring.

**Table 1:** Biometric parameters, symptom questions, and alert triggers

**Biometric parameter**	**Urgent alert**
Systolic blood pressure with symptoms	SBP < 90 mm Hg *or* > 160 mm Hg
Systolic blood pressure without symptoms	SBP < 80 mm Hg *or* > 170 mm Hg
Heart rate with symptoms	HR < 50 *or* > 100
Heart rate without symptoms	HR < 40 *or* > 110
Weight with symptoms	Daily gain > 3 lbs *or* weekly gain > 6 lbs
**Symptom questions – Group 1**	
Have you felt more short of breath in the last day?	Yes
Have you noticed more swelling in the last day?	Yes
Have you had any light-headedness or dizziness in the last day?	Yes
**Symptom questions – Group 2**	
Did you wake up short of breath last night?	Yes
Did you sleep in a chair or propped up with pillows, more than usual last night?	Yes
Compared to yesterday, would you say you are feeling about the same, better, worse, or much worse?	Much worse

‘With symptoms’ is determined based on responses to symptom questions, or ascertained in telephone conversation with the patient.

### Control

After randomization, the site nurse gives patients in the usual care (control) arm a study binder that contains a one page summary of the study, a checklist for completion of the 7-day, 30-day, and 180-day telephone surveys, a copy of the upcoming 7-day survey, and copies of their consent and HIPAA authorization forms. Control patients have no further contact with site study nurses or call center nurses. However, they may be exposed to other readmission reduction or chronic disease management programs implemented by hospitals, physician groups, or health plans, such as education about heart failure, pharmacist consultation, and post-discharge telephone calls. We collected information about usual care at each of the six sites at the beginning of the study and will continue to do so during the study, so that we will be able to describe how the usual care transition for patients with heart failure has evolved over time.

### Study measures

The primary outcome measure is the 180-day all-cause readmission rate. Secondary outcomes are the 30-day readmission rate, mortality, ED visits, hospital days, hospital costs, and HRQOL. These are collected at the time intervals shown in Table 2. Costs will be analyzed from both societal and provider perspectives. HRQOL will be measured by the Minnesota Living with Heart Failure Questionnaire (MLHFQ) [23]. Table 3 provides a complete list of variables collected, means of measurement, and timing. The Total Illness Burden Index [24] will augment co-morbidities obtained from hospital administrative data. Sociodemographic measures include employment and productivity questions from the Health and Work Performance Questionnaire [25]. Other measures of interest include the brief Care Transition Measure (CTM-3) [26], the Rapid Estimate of Adult Literacy in Medicine, Revised (REALM-R) [27], the Geriatric Depression Scale [28], heart failure self-care knowledge, behaviors, and confidence from the Self-Care of Heart Failure Index (SCHFI) [29], the Lubben Social Network Scale [30], questions about informal caregiving [31], and the Morisky Medication Adherence Scale [32]. In addition to the adherence measures included in the survey questionnaire, we will measure adherence to the overall intervention and its components using data transmission records and telephone call documentation. The final (180-day) survey also includes questions about satisfaction with the overall intervention, the post-discharge nurse component, and the post-discharge device component.

**Table 2:** Outcome measures

**Time frame**	**Readmission rate**	**Mortality**	**Emergency department (ED) visits**	**Hospital use**	**Hospital costs**	**Quality of life**
Initial hospitalization		Inpatient death		Index length of stay	Total hospital costs	MLHFQ score
30 days after index discharge	30-day readmission rate	30-day mortality rate	ED visits within 30 days	Total hospital days	Aggregate hospital costs	MLHFQ score
180 days after index discharge	180-day readmission rate	180-day mortality rate	ED visits within 180 days	Total hospital days	Aggregate hospital costs	MLHFQ score

MLHFQ is Minnesota Living with Heart Failure Questionnaire.

**Table 3:** Source and schedule of outcome, utilization, process, and other variables

			**Post-discharge**		
**Variable**	**Source/Instrument**	**Baseline**	**7-days**	**30-days**	**180-days**
***Outcomes***					
Readmission	UHC, OSHPD		X	X	X
Hospital days	UHC, OSHPD		X	X	X
Hospital cost	UHC, OSHPD				
Emergency Dept use	OSHPD		X	X	X
Mortality	UHC, OSHPD, National Death Index		X	X	X
Quality of life	Survey/Minnesota Living with Heart Failure Questionnaire	X	X	X	X
***Intervention process***					
Pre-discharge education completion and comprehension	Enrollment documentation notes	X			
Post-discharge health coaching	Call center documentation notes		*	*	*
Remote monitor use	Daily data transmission reports		*	*	*
Calls triggered by remote monitoring	Data transmission reports, call center documentation notes		*	*	*
Patient assessment of care transition	Survey/Care Transition Measure-3		X		
***Covariates***					
Age	Survey, UHC	X			
Gender	Survey, UHC	X			
Race/Ethnicity	Survey, UHC	X			
Language	Survey, language of consent	X			
Household income	Survey	X			
Education	Survey	X			
Marital status	Survey	X			
Insurance (for example, dual Medicaid/Medicare)	UHC	X			
Employment	Survey/Health and Work Performance Questionnaire	X		X	X
Health literacy	Survey/REALM-R	X			
Severity of illness	Survey/Total Illness Burden Index	X			X
Co-morbidities	UHC	X			
Depression	Survey/Geriatric Depression Scale	X	X	X	X
Self-care behaviors	Survey/Self-Care of Heart Failure Index	X	X	X	X
Social networks	Survey/Lubben Social Network Scale	X		X	X
Informal caregiving	Survey/Medical Care Questionnaire	X		X	X
Medication adherence	Survey/Morisky Medication Adherence Scale		X	X	X
End-of-life wishes	Survey	X			

*Signifies the measure is continuously available over the 180-day timeframe.

UHC is University HealthSystem Consortium; OSHPD is Office of Statewide Health Planning & Development.

### Data collection

Same-hospital readmissions and hospital days will be obtained from administrative data routinely submitted by the study hospitals to the University HealthSystem Consortium (UHC), a voluntary association of academic medical centers to which all six participating medical centers belong. ED visits to the study hospitals as well as readmissions, hospital days, and ED visits to other California hospitals will be obtained through linked inpatient discharge and ED data from the California Office of Statewide Health Planning and Development (OSHPD), submission of which is mandated by state law. Hospital costs for the study hospitals will be obtained from the UHC data, while costs for admissions and ED visits to non-study hospitals will be estimated from the OSHPD data.

A baseline survey is conducted face-to-face by the hospital study nurse prior to randomization. Patient reported data are collected by telephone surveys conducted at 7 days, 30 days, and 180 days after the patient’s initial hospital discharge. The telephone survey interviewers are blinded to the respondent’s randomization status. Study patients are given a paper copy of the 7-day survey as part of their enrollment packet and receive advance copies of each upcoming survey by mail. They receive a $10 gift card in the mail following completion of each telephone survey.

A Data and Safety Monitoring Board (DSMB) is overseeing the conduct of the study. The committee consists of three researchers from universities not involved in the study. Two members of the UCLA Department of Medicine who are not involved in the study convey data from the study team to the DSMB. Adverse event reports are completed for patient readmissions and deaths and reviewed by the DSMB, with the primary objective of ascertaining any delays in care that occur because patients rely on being monitored by the study.

### Statistical analysis

Our analytic approach will use multivariate regression analysis to compare study outcomes between the intervention and usual care groups, adjusting for patient characteristics that can influence resource use and mortality. These analyses will use an intent-to-treat (ITT) framework, within a hierarchical approach using information on medical centers and patients.

Confounding will be assessed by comparing the unadjusted coefficient for treatment condition with the adjusted coefficient. Several of the resource use variables, such as trigger calls and total 180-day hospital readmissions, will likely have non-normal distributions with high skew. As in previous analyses [6], we will draw upon statistical approaches and models developed for handling this type of data, such as transformation for non-normal distributions; two-part models to handle zero values and skewed non-zero values separately; split-sample techniques to distinguish between different functional forms and to avoid overfitting; and count models (for example, Poisson and negative binomial models). We will confirm model selection with goodness of fit tests. Although BEAT-HF is not a cluster randomized trial, there is potentially non-random clustering of patient characteristics occurring at the six study sites because we randomized within hospitals. As a result, our quantitative analyses will use mixed effects hierarchical linear models, with two-level models for analyses of patients nested within medical centers, and three-level models for analyses of repeated measurements on patients nested within medical centers.

### Sample size

Sample size was calculated based on the assumption that the control group would experience no change in the observed baseline 180-day readmission rate of 38%. A sample size of 1,500 (750 per arm) will provide 80% power to detect a relative reduction of 28% in the primary outcome with a significance level of 0.05, after adjusting for within-hospital clustering. We expect to screen approximately 31,500 admissions in order to enroll 1,500 patients. Patients who are readmitted after having given a firm refusal are not asked again, but those with a ‘soft’ refusal are approached again if readmitted.

## Discussion

The BEAT-HF approach differs from telemonitoring programs previously described in several ways. It incorporates elements of successful care transition programs by engaging patients during their hospitalization and combining centralized structured telephone support with telemonitoring. Tele-HF was classified as a structured telephone support intervention, because it used an interactive voice response system that requires patients to enter information such as weight and blood pressure on a standard telephone keypad, but the ‘interaction’ was between the patient and a recorded voice. In BEAT-HF, the telephone is used for a coaching interaction between patient and nurse. In Tele-HF, monitoring data were sent directly to a cardiologist affiliated with the recruitment site, not the patient’s regular physician. The BEAT-HF nurses first call the patient in response to an alert trigger, enabling them to screen out false readings and to assess the severity of the patient’s symptoms. They also interact by telephone with patients’ regular providers, communicating the trigger in its clinical context and reinforcing existing patient-provider relationships by providing additional information about their patients’ needs.

Conducting transition coaching by telephone enables the nurses to support significantly more patients than if they were traveling to patients’ homes. Centralizing the nurses enables them to serve patients discharged from multiple hospitals, including those where the volume of heart failure patients would not be sufficient to employ a full-time nurse efficiently. The issue of scale is important because the prevalence of patients with heart failure may be too great to accommodate them in comprehensive disease management programs that rely on specialized clinics or home visits. In addition, some patients are too frail to make regular visits to outpatient clinics [33,34]. The initial follow-up for monitoring triggers is performed centrally, assuring a uniform response. Finally, combining nurse coaching with telemonitoring allows the nurse to address issues related to patient use of the equipment.

Data captured as part of BEAT-HF will enable us to elucidate some of the reasons for non-adherence, a problem experienced by Tele-HF and other studies. These reasons may include real or perceived equipment failure or lack of reliability, patients who travel, patients who are admitted to skilled nursing or readmitted to a hospital, as well as behavioral non-adherence. This information will be extracted from the call logs maintained by the call center nurses and Ideal Life service representatives. Some of the technical difficulties may be due to human factors issues.

It is likely there will be variation in the ‘dose’ of intervention received by patients over the 180 days of the study in terms of both their adherence to telemonitoring and acceptance of coaching telephone calls. The primary outcome will be analyzed on an intention-to-treat basis. However, it also will be important to evaluate whether there is a dose-response effect. The ‘dose’ of telemonitoring will be captured automatically by the data transmissions. The dose of nurse coaching is affected not only by patients’ willingness to accept calls, but by the number of alert triggers generated by the patients’ transmitted parameters. The quantity of triggers may be associated with a patient’s severity of illness, but it may also reflect persistently high or low blood pressure or heart rate, or technical difficulties with the equipment.

The BEAT-HF study has other strengths. It is larger than most care transition or telehealth studies for heart failure, and may be the only large U.S. study to include a substantial proportion of patients with limited or no English proficiency. The patients are treated in different systems of care, with medical centers ranging from a safety net provider for a large, mixed urban-rural area to a major urban hospital that incorporates elements of both academic medicine and a large private practice medical staff.

At the same time, the study faces several challenges. The ascertainment of patient readmissions and ED visits is key. Because approximately 20% of readmissions occur to other (non-study) hospitals, and patients may not remember hospitalizations or ED visits, complete determination of utilization will depend on the success of matching patient identification data with OSHPD data.

Rapidly changing telecommunications technology led to technical problems at the beginning of the study. The Ideal Life equipment deployed initially used a modem that required landline telephone service to transmit data. However, a growing number of households no longer have traditional telephone service because they use a subscription or prepaid mobile phone exclusively, or they have bundled television, internet, and telephone service which disrupted landline data transmission. In response, Ideal Life introduced the Pod™, which uses any available cellular signal. Technology issues have led both research staff and Ideal Life representatives to dedicate more time than planned to individual patient trouble-shooting and home visits and increased the number of contacts between the call center nurses and some patients.

This study is taking place during a time of significant change in hospitals’ financial and regulatory environment. Reducing readmissions plays a key role in each of the top three priorities identified by hospital CEOs responding to an annual survey conducted by the American College of Healthcare Executives: financial concerns, patient safety and quality, and implementing health reform [35]. To the extent possible, it will be important to identify other disease management initiatives underway at the participating sites and to assess their effect on both control and intervention patients [36]. We recognize that other initiatives may create an environment that is particularly responsive - or unresponsive - to telemonitoring and patient education.

Despite our best efforts to recruit a sample that is representative of the target population of older adults with heart failure, our results may have limited generalizability. In particular, our sample will inevitably under-represent some of the highest risk, most vulnerable patients in our communities: those housed long-term in skilled nursing facilities, those without a usual source of medical care, those without insurance or with limited Medicaid coverage, those who require chronic renal dialysis, those with moderate-to-severe cognitive impairment, those who are too functionally impaired to use a scale and a telephone, and immigrants who speak only Asian or other languages not used in the study. However, it may be inevitable that system interventions, such as those studied in BEAT-HF, can only be delivered in certain settings, and must be adapted before they can be delivered in other settings or to other populations.

BEAT-HF is one of the largest randomized controlled trials of a telehealth approach to improving outcomes for patients with heart failure. It incorporates elements of successful care transition programs, combining centralized structured telephone support provided by nurses with home telemonitoring using the latest remote monitoring technology. BEAT-HF is enrolling patients with a wide range of socioeconomic and demographic backgrounds and collecting extensive data on intermediate factors that potentially affect patient adherence. As a result, the study will provide a wealth of information on how different individuals use technology and respond to interventions that are not face-to-face encounters. Once completed, BEAT-HF is poised to serve as an important research resource to understand how best to use telehealth approaches to improve key healthcare processes and outcomes, including care transitions and hospital readmissions, and to set the stage for future comparative effectiveness research on chronic disease management for heart failure.

## Trial status

At the time of manuscript submission, the BEAT-HF study is actively enrolling participants.

## Competing interests

The authors declare that they have no competing interests.

## Authors’ contributions

MO is the overall Principal Investigator (PI) of the study and obtained funding. AA, JB, TG, SG, and PR are site PIs overseeing implementation of the protocol at their respective sites. MO, AA, JB, TG, SG, SK, and PR contributed to the study design. MO was primarily responsible for the analysis plan. JB wrote the manuscript with contributions from BS, PR, TG, SG, SK, and MO. All authors read and approved the final manuscript.
